# West Nile Virus Antibodies in Colombian Horses

**DOI:** 10.3201/eid1109.050426

**Published:** 2005-09

**Authors:** Salim Mattar, Eric Edwards, Jose Laguado, Marco González, Jaime Alvarez, Nicholas Komar

**Affiliations:** * University of Córdoba, Montería, Córdoba, Colombia;; † Centers for Disease Control and Prevention, Fort Collins, Colorado, USA

**Keywords:** West Nile virus, Colombia, Latin America, horse, arbovirus, flavivirus, letter

**To the Editor:** West Nile Virus (WNV) is rapidly spreading in the Western Hemisphere (*1*). We report the first evidence for WNV transmission in South America.

WNV is serologically related to the Japanese encephalitis complex of flaviviruses (Flaviviridae), which includes Saint Louis encephalitis virus (SLEV) (in North and South America), Japanese encephalitis virus (Asia), and Murray Valley encephalitis virus (Australia) (*2*). Because of antigenic cross-reactivity within this complex, WNV serologic diagnosis requires highly specific assays, such as the plaque-reduction neutralization test (PRNT) (*3*). We used PRNT to evaluate serum collected from 130 healthy equines (horses and donkeys) in Colombia, where WNV had not been previously reported. These equines were sampled between September 15 and October 29, 2004, in the northern departments of Córdoba and Sucre in the Caribbean region of Colombia. Samples were heat-inactivated and titrated by PRNT for antibodies to WNV, SLEV, and 3 other South American flaviviruses: Rocio, Ilhéus, and Bussuquara. Twelve specimens (9%) from 10 different premises tested positive for WNV (Table). None of these animals had been vaccinated against WNV or had traveled outside of the region. An equine immunoglobulin (Ig) M-capture enzyme-linked immunosorbent assay (ELISA) that used WNV antigen detected anti-WNV IgM in 2 of the 12 specimens, which indicated that some of these infections were relatively recent (probably within 3 months of sampling). The positive findings in both Córdoba and Sucre were corroborated by a WNV-specific blocking ELISA (*4*). Numerous other samples exhibited flavivirus reactivity in the neutralization and blocking ELISA assays, mostly because of SLEV. Complete test results from these horses, as well as from Colombian cattle and chickens, will be presented elsewhere.

**Table:** PRNT90 antibody titers to WNV and other South American flaviviruses for Colombian equine sera*†

Equine ID‡	Department	Age (y)	WNV	SLEV	ILHV	ROCV	BSQV
3	Córdoba	4	1:40	<1:10	<1:10	<1:10	<1:10
35	Córdoba	5	1:160	<1:10	<1:10	<1:10	<1:10
39§	Córdoba	4	1:40	<1:10	<1:10	<1:10	<1:10
41	Córdoba	6	1:40	<1:10	<1:10	<1:10	<1:10
48	Córdoba	4	1:640	<1:10	<1:10	<1:10	<1:10
76	Sucre	5	1:80	<1:10	<1:10	<1:10	<1:10
85	Sucre	9	1:80	<1:10	<1:10	<1:10	<1:10
94	Sucre	3	1:40	<1:10	<1:10	<1:10	<1:10
101	Sucre	4	1:40	<1:10	<1:10	<1:10	<1:10
109§	Sucre	7	1:160	1:40	1:40	1:10	<1:10
123	Córdoba	6	1:40	<1:10	<1:10	<1:10	<1:10
125	Córdoba	4	1:160	<1:10	<1:10	<1:10	<1:10

*These 12 specimens were considered positive for WNV infection; a 4-fold WNV PRNT_90_ titer compared to that of other flaviviruses was required for a positive determination of previous WNV infection. †PRNT_90_, 90% plaque reduction neutralization test; WNV, West Nile virus; SLEV, Saint Louis encephalitis virus (South American strain); ILHV, Ilhéus virus; ROCV, Rocio virus; BSQV, Bussuquara virus. ‡All equines were horses except for 76 and 85, which were donkeys. §Also positive for anti-WNV immunoglobulin M by antibody-capture enzyme-linked immunosorbent assay.

These serologic data should be considered indirect evidence of WNV activity in Colombia. We encourage Colombian human and animal health authorities to enhance surveillance for human, equine, and avian disease attributable to WNV. Efforts are needed to isolate the virus or detect specific viral RNA to confirm this finding and to identify vectors and vertebrate hosts involved in WNV transmission in Colombia.
